# Patch-Based Ray Tracing in NanoShaper Boosts Molecular Surface Computation

**DOI:** 10.1021/acs.jcim.5c02287

**Published:** 2025-12-23

**Authors:** Marco Domenico Mazzeo, Vincenzo Di Florio, Walter Rocchia, Sergio Decherchi

**Affiliations:** † Concept Lab, 121451 Fondazione Istituto Italiano di Tecnologia, via Morego 30, 16163 Genoa, Italy; ‡ MOX Laboratory, Department of Mathematics, Politecnico di Milano, Piazza Leonardo Da Vinci, 32, Milano 20133, Italy; § Data Science and Computation Facility, 121451 Fondazione Istituto Italiano di Tecnologia, via Morego 30, 16163 Genoa, Italy

## Abstract

NanoShaper (NS) is a widely used tool that leverages ray tracing for molecular surface triangulation, pocket detection, and supports Poisson–Boltzmann equation solvers. By retaining the established methodological framework and implementing a targeted redesign, our approach achieves performance gains of up to 12.5× and memory reductions of up to 8×, enabling the triangulation of complexes containing millions of atoms on relatively modest computational architectures in a short time. The key innovation is a patch-based ray tracing algorithm that replaces the traditional ray-sweeping approach. By iterating over surface patches rather than rays, this method enhances cache performance and removes the need for memory-intensive grids for patch localization, yielding major reductions in memory usage. Further optimizations include the parallelization of the analytical solvent-excluded surface (SES) construction and the replacement of uniform grids and octrees with bilevel grids and/or compressed buffers. We also introduce an analytical ray–torus intersection scheme based on an exact quartic solution, which improves both accuracy and computational efficiency. The updated version (v1.5) additionally provides a public C++ API for seamless integration with external tools. The tool is available at https://gitlab.iit.it/SDecherchi/nanoshaper. Results on more than 1500 structures and on a multimillion atom complex (e.g., the H1N1 virus) confirm speed and accuracy achievements.

## Graphical abstract

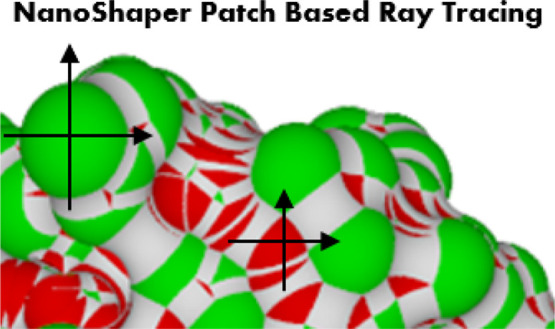

## Introduction

Computing the molecular surface (MS) is a fundamental task in atomistic modeling, with applications ranging from biophysical analysis to drug discovery, including pocket detection and druggability assessment.^1,2^ A precise definition of the molecular surface is particularly critical in the context of continuum electrostatics, where it defines the boundary between solute and solvent domains, and governs the spatial distribution of dielectric properties, directly influencing the numerical solution of the Poisson–Boltzmann equation.

Several tools exist for molecular surface generation^3,4^ and NS^5,6^ has demonstrated high efficiency and accuracy in producing analytically defined triangulations of molecular surfaces, as confirmed in several applications.^7,8^ Some features are distinctive to NS and distinguish it from other methods. NS systematically utilizes an analytical approach: for instance, the buildup of the SES follows both a numerically and structurally rigorous route. The geometrical constructs employed bear an accuracy which goes beyond the double precision by leveraging the CGAL library. This is essential to robustly build the regular triangulation complex, where double precision alone may fail. Second, all the stages of the NS’s workflow are, in principle, intrinsically trivially parallel; this renders its theoretical scalability very favorable. Specifically, NS follows a four-stage pipeline: (i) analytical construction of the surface (e.g., SES patches), (ii) ray tracing (RT) to sample in/out information on a regular grid, (iii) grid coloring and volume estimation, (iv) application of the Marching Cubes (MC) algorithm to extract a triangulated mesh from the intersections data (see Figure 1) followed by mesh smoothing and file saving. This algorithmic approach offers both analytical precision and a high level of parallelism. In the previous public release (v0.8), the surface construction phase is not parallelized, whereas the RT and MC stages are.

**Figure 1:**
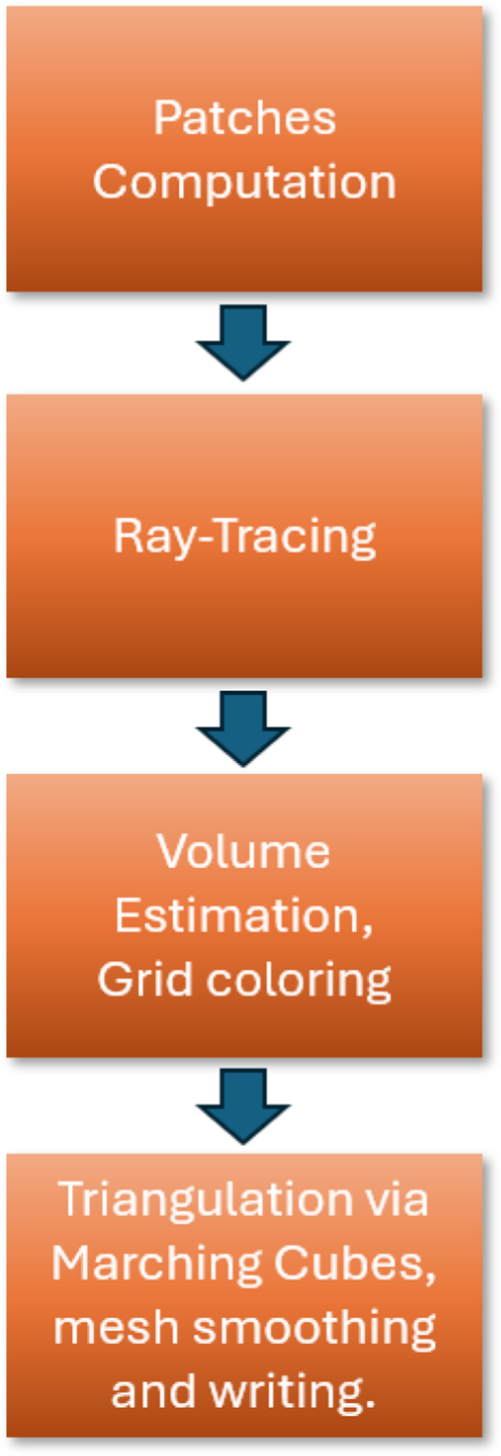
NS main computational steps are (a) computation of the equations and trimming solids of the surface patches, (b) axis-aligned ray tracing of the set of patches, (c) estimation of the volume integrals and determination of the surface in/out information, and (d) triangulation via the Marching Cubes, mesh smoothing, and saving.

Additionally, NS is able to compute pockets.^6^ A pocket is defined as a region of space which is accessible by water but not accessible by a *bigger* molecule. To implement this principle we compute two SESs, one is the regular one with the probe radius emulating water, while the second is still a SES but employing a bigger probe, 3.0Å by default. We get the set of pockets by taking the volumetric grid-based difference of the big SES and the regular SES. These pockets are then isolated (as the initial differential volumetric map is unique) and each one triangulated. The pockets isolation is supported by a recursive application of a flood filling algorithm on the differential grid. The triangulation of the pockets is supported by placing virtual probes (virtual atoms) on the grid points which identify the pockets; these are then triangulated by approximating the surface as the Skin Surface of the virtual probes. This allows to get a set of triangulated pockets.

Last, NS is available via a web server at https://nanoshaperweb.iit.it;^9^ Figure 2 shows an exemplary surface on which a pocket identified by NS is specifically visualized via the web server viewer.

**Figure 2:**
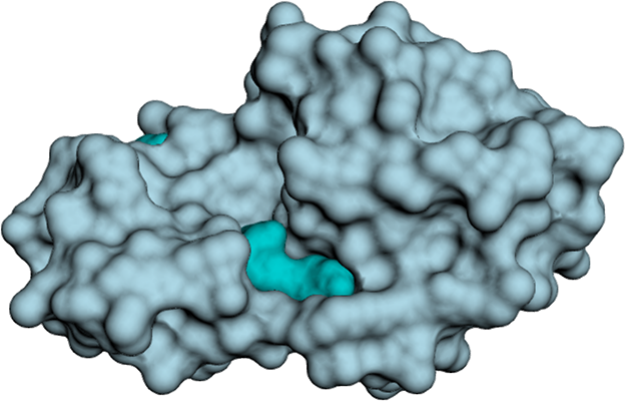
Exemplary NS surface (pdb id: 2RBN) computed by NS and a detected pocket visualized via the Nanoshaper web server (https://nanoshaperweb.iit.it).

All the previously described features are already supported by the previous version of NS, version 0.8 (we will call it *previous version* throughout the text). In this work, we introduce a major update of NS, version 1.5 (we will refer to it as *new version*), which is capable of both drastically reducing memory usage and decreases execution time. The central innovation is a patch-based ray tracing algorithm, which replaces the traditional rays-sweeping approach and eliminates the need for grid-based patch localization. This novelty allows us to obtain substantial memory savings, and hence to triangulate larger structures. In addition, we parallelized the SES construction phase and carried out several optimizations, including bilevel grids, compressed buffers and an analytical ray–torus intersection solver. All the new features can be enabled/disabled via new configuration keywords documented in the manual. In the following sections we discuss the algorithmic and technical improvements, and then we present the corresponding computational results.

## Parallel Solvent-Excluded Surface Buildup

Here, we describe the parallelization strategy used to accelerate the build-up phase of the SES. This phase refers to the process that defines the SES patches and their associated trimming solids. This begins with the computation of the alpha shape complex (a filtration of the regular triangulation), from which patch parameters and trimming solids are derived.^5^

In the previous version of NS, v0.8, the alpha shape complex (instrumental to define patch parameters) is built using CGAL;^10^ latest versions of this library can run this task in parallel. However, this approach does not fully exploit the inherent parallelism of all the build-up tasks. Since the SES construction is spatially local, as each patch and its trimming solid solely depend on neighboring atoms, it can naturally be addressed by a domain decomposition strategy, enabling concurrent patch generation.

In the approach we now employ, the domain is partitioned along the Y axis into *N* slabs, each processed by a separate thread, independently of the others (see Figure 3). There is no particular reason for choosing the Y axis, instead of the other ones; we here recommend to preprocess very anisotropic structures to align the main inertia axis of the system with the Y axis so as to enhance multithreading during the use of NS. Given this decomposition we build a local alpha-shape for each subdomain (subset of atoms), rendering the parallelism fully explicit. Slab boundaries are chosen so that each one contains approximately the same number of atoms to ensure balanced workloads. This is achieved utilizing a fine-grained binning method: atoms are first counted in narrow bins (dubbed ”mini-slabs”), and then slab boundaries are determined to render atom counts across threads as similar as possible.

**Figure 3:**
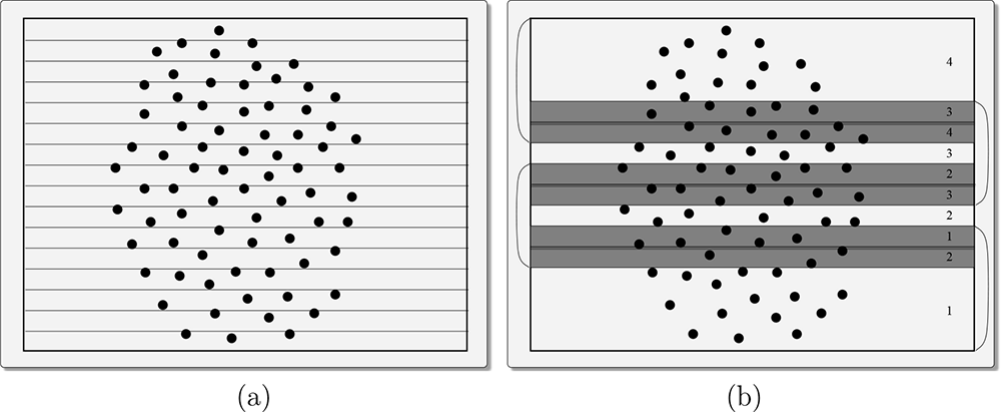
(a) Domain is first subdivided into fine-grained bins along the Y-axis, and atoms (black dots) are counted in each bin. (b) Bin counts are analyzed to define four slabs (corresponding to four threads), with boundaries chosen to ensure an approximately equal number of atoms per slab. Slab indices are labeled within the light gray regions. Dark gray bands represent halo layers, which provide neighboring atomic data needed for correct patch construction across slab boundaries. Halo regions are labeled with the index of the slab they belong to. Brackets on the sides visually group each slab and its associated halo layers. Note that the outermost slabs (top and bottom) are wider than the central ones, reflecting the nonuniform atom density in the domain.

Each thread processes the atoms within its slab and also those in adjacent *halo layers* to have correct results, as shown in Figure 3, which account for interslab patch dependencies. These halo/boundary layers ensure that the surface in a slab is correctly built as all atoms, which participate to the determination of the patches, including the ones outside the current slab, are involved. The required halo layer thickness is determined by the probe radius, as larger probes produce broader patches. We found out that, commonly, a 9 Å halo layer thickness is sufficient for typical probe sizes (≤2 Å) (see the results section) to get fully quantitative consistency with single-thread results. We found, however, that on a huge system (virus) 11 Å was needed to get a very accurate matching with the serial result. Consequently, conservatively, each slab must be at least 12 Å thick, which imposes an upper limit on the number of usable slabs (and thus threads) for a given molecular system. If the requested number of threads exceeds this limit, it is capped accordingly.

After this phase, duplicated patches—those that span slab boundaries—are identified and removed. A patch is discarded if the bottom-most vertex of its trimming solid lies outside the horizontal bounds of the thread’s assigned slab. This filtering ensures a uniquely defined set of SES patches, which are then passed to the ray tracing stage.

## From Conventional to Patch-Based Ray Tracing

In this section, we describe the original ray tracing (RT) method used in NS and how we restructured it to achieve significant improvements in performance and memory savings by leveraging specific features of the molecular surface computation problem.

### Conventional Ray Tracing

In standard RT, rays are cast from a light source, and their interactions with surface elements such as reflections, refractions, or occlusions are tracked. In general-purpose graphics, this often requires complex acceleration structures (e.g., bounding volume hierarchies, BVHs^11^) to efficiently identify surface intersections.

NS uses a specialized form of RT tailored to its task. While traditional RT is primarily aimed at producing 2D visualizations, the NS’s method conducts a sort of full 3D RT: rays traverse the spatial domain to directly sample the surface geometry, rather than terminating at the first surface hits. Rays are cast from all the three axis-aligned planes (XY, YZ, XZ), and the molecular surface is considered fully transparent: all intersections along each ray must be detected. The molecular system is embedded in three regular 3D grids, one for each coordinate plane, wherein the indices of the patches spatially spanning a pixel are stored in an appropriately long array. Rays are cast from *pixels*, defined as the grid points lying on the currently considered bounding cube face and coordinate plane. Each pixel casts a single axis-aligned ray through the volume.

Since multiple intersections (not necessarily ordered) along a ray are present, these are first collected, then sorted along the ray’s direction. This sorted list is used to assign in/out status to grid cubes via a standard parity-check.

### Patch-Based Ray Tracing

The previous ray-centric approach iterates over rays and requires spatial acceleration structures to efficiently locate pierced patches along their paths. However, one important observation enables a more efficient strategy in our context: every (nondegenerate) surface patch will be intersected by at least one ray, and no patch occludes another. In principle, there may exist patches which are not intersected by any ray, however this is an unusual case as it would mean that the grid is very coarse with respect to the molecular geometry, hence our previous assumption is very likely to hold. This insight allows us to invert the logic: instead of iterating over rays and querying for patch intersections, we directly iterate over patches and determine the set of piercing rays (see Figure 4). The procedure is as follows:

- For each patch, calculate the bounding box of its trimming solid.
- From the bounding box, identify the set of rays (along each of the three directions) that pass through it.
- Compute all intersections between the patch and these rays.
- Store intersections in a ray-based fashion using the ray pixel identifiers. Sort the intersection points along the rays.
- Check the parity of intersections to identify the in/out status of grid cells.

**Figure 4:**
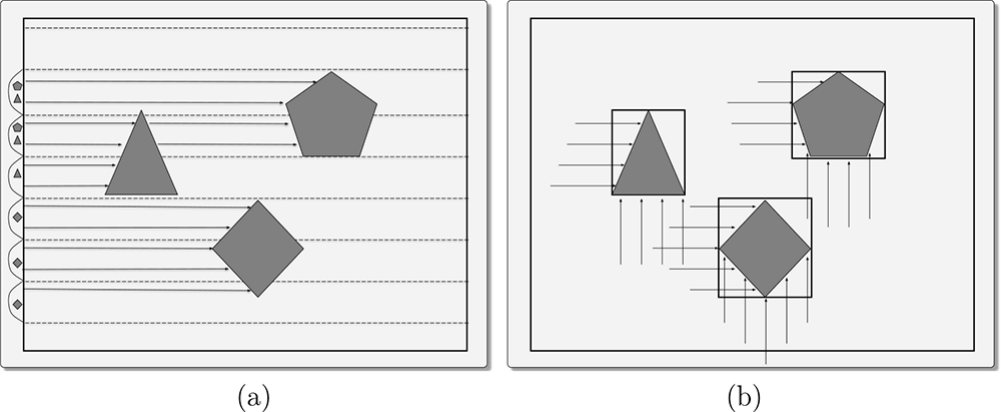
(a) Conventional ray tracing: rays are cast from the light source (axis-aligned planes in our case), sampling the volume enclosed in the MS. (b) Patch-based ray tracing: all rays intersections are collected for every single patch. Next, the procedure iterates over all patches. By reversing the iteration logic from rays to patches, one completely avoids spatial indexing overhead and significantly improves cache usage.

This patch-based ray tracing eliminates the need for spatial acceleration structures entirely, yielding significant memory savings. Moreover, since all the ray directions are processed simultaneously for each patch, this method achieves better CPU cache utilization and improved data locality compared to the ray-centric RT approach. When treating large molecular complexes, the ray-centric technique often requires storing numerous unordered patches per pixel, which can lead to significant cache trashing and large memory usage. This issue becomes particularly pronounced when neighboring pixel vectors contain long lists of patch identifiers, as every patch occupies several tens of bytes in memory, resulting in greatly scattered data and severe cross-patch memory striding even when the data sets are not large. The patch-based scheme may resemble the in situ visualization technique implemented in^12^ in which, during a parallel simulation, each MPI task is devoted to the simulation of the subdomain initially assigned to it and its rendering, simultaneously; each simulation screenshot relies on a parallel building of the full image starting from the partial per-task subimages. In situ visualization and analysis, and our patch-based technique are totally different with regard to objectives: the former aims at sidestepping time-consuming I/O stages, especially when dealing with large data sets and several time instances of interest, whereas the latter focuses on optimizing cache usage and memory space, as previously discussed.

### Analytical Torus-Ray Intersection

The previous NS version adopts a robust iterative procedure based on the Sturm method^5,6^ for determining ray-torus intersections. As previously discussed, in NS an in/out parity-check along each ray is carried out to determine whether a grid point is inside the surface or not. Indeed, by construction, rays start and finish outside the surface. As a consequence, every ray must have an even number of intersections. If this does not hold true, one or more intersections are missed and/or computed inaccurately. If NS detects such an issue, it copies the data spatially corresponding to the previously processed ray into the data of the current ray. This generates a locally constant manifold surface, which is then triangulated in a consistent manner. The main cause of these “failed” rays is the imperfect ray-torus intersection computation. Despite the fact that the final surface is still manifold, and that these cases are rather infrequent, their presence introduces an approximation. As the ray-torus intersection boils down to a quartic polynomial root-finding problem, the new NS version replaces the iterative approach with an analytical one. The numerical root finding of quartic and cubic polynomials, seemingly simple, needs significant numerical care.^13,14^

Given the polynomial in the quartic form, *x* ^4^ + *bx* ^3^ + *cx* ^2^ + *dx* + *e* = 0, the sign of its discriminant is employed to determine how many of the roots are real. In the particular case in which all the coefficients of the linear and quadratic terms or the discriminant are exceedingly large, and provided that the constant term of the original quartic is not zero, we perform the change of unknown from *x* to *x̂* = 4*e* (*x*–*d*). The procedure borrows from the original Ferrari’s analytical method to solve quadratic polynomial equations, with an ad hoc fix of possible divisions by zero or by small numbers. It first calculates the largest positive solution of the associated cubic polynomial equation, *m*, basically following the Cardano’s procedure. Also for the cubic equation, critical cases arise when the coefficients of the linear or of the constant parts are much smaller than that of the quadratic term, and both the usage of complex numbers and the division by coefficients that could become infinitesimal or null are avoided by exploiting the symmetry of the solutions (see the pseudocode of the procedure in the Supporting Information (SI)). Once *m* is found, the Ferrari’s procedure can be completed, distinguishing the case when *m* is close to zero. Accuracy of these solutions, tested on thousands of intersections on different molecular systems and calculated against the companion matrix method (by means of Matlab^15^), showed an absolute error greater than 10^–8^ in less than the 0.09*%* of the cases. The new approach reduces the number of failed rays and compute time, as later shown in the results.

## Further Optimizations

### Optimized Storage

During the ray tracing process, NS resorts to octrees to store intersections and other information.^6^ Octrees are convenient in atomistic systems since wide spatial regions are occupied by the solvent and are free of ray-surface intersections. We replaced octrees with bilevel grids to further improve the storage requirements. In this data structure the first level grid is a uniform coarse one bearing a 4-time reduced resolution with respect to the desired one. These big-grid cubes are made empty by default, except for a null pointer inside. Solely in the necessary case in which there is a surface boundary crossing the grid we allocate (inside these big-grid cubes) a small uniform grid containing 4^3^ smaller cubes of a resolution which matches the desired one. The RT stage allocates and updates the small grids only where necessary. In this context, bilevel grids are advantageous with respect to octrees, or other deep trees, for multiple reasons. Deep trees well adapt to sparse, multiscale and largely inhomogeneous data distributions. However, they incur in unnecessary memory overhead for sparse but uniformly resolved data, as in the current case. This happens as numerous subtrees are without leaves and do not store useful data. Moreover, deep subtrees require slow many-step data accesses to descend them. These issues are exacerbated in the presence of large data sets. Having instead 4^3^ voxels per small grid guarantees a compact and efficient data layout. Data retrieval is optimized by employing power-of-two integer numbers (see SI for further details). After multithreaded patch piercing, the coloring of the grid with the in/out status is done in parallel. Threads are interleaved along the *Y* and *Z* axes, depending on the ray directions, which correspond to the leading buffer dimensions so as to provide a cache friendly and load balanced pattern.

Arrays of booleans (or integers requiring a few bits) are compressed by taking advantage of bits inside 32-bit words to optimize memory. The previous NS version resorts to three octrees for storing vertices’ indices along the three coordinates plus three octrees for storing their corresponding normals. First, we substitute octrees with bilevel grids. Second, we now use only three bilevel grids instead of six to index vertices and normals jointly. As shown in the results section, the aforesaid post-RT assembling stage, referenced later as “Assembling,” is drastically faster when the new code version is used.

After determining vertices and triangles, a Laplacian smoothing stage can be carried out to adjust the position of the vertices. Each vertex is slightly displaced by taking the average coordinates of its neighboring vertices; every new normal is computed similarly. The previous NS version allocates the neighbor list of every vertex and fills it by looping over the vertex indices of every triangle and using the intratriangle indices’ subset to contribute to the filling of the neighbor lists. Also, it allocates and updates the per-vertex neighbor counts and the new per-vertex positions and normal components. Before adding a potentially new neighbor vertex index to a reference vertex, the current status of its neighbor list is checked to avoid including it twice. The new NS version, instead, does not allocate the neighbor lists and while looping over the intratriangle indices, it updates the neighbors’ counts without performing that check: since the surface is closed, one knows a priori that any triangle edge is shared by two triangles, and hence that every count, vertex and normal value sum is doubled. Consequently, the positional and normal contributions stored in the aforesaid extra new vertex and normal buffers are to be simply divided by twice the values registered in neighbor counts’ buffer. This results in a faster and memory cheaper smoothing stage, as shown in the results section.

### Triangulation and Normals Calculation

We optimized some loops and buffers useful to the MC stage to more profitably exploit processor caches. Also, we compressed the boolean grids by storing any contiguous 32-boolean-value group in one unsigned integer.

From the numerical stand point the ray-patch intersection routines may incur in approximation errors. Consequently, it may sporadically occur that intersections are not detected or correctly computed. Rarely, it may also occur that two consecutive intersections are very close to each other and occupy the same MC cube edge; in the SI we provide values quantifying these sources of inaccuracy. Also, when one intersection is missed, the number of intersections along the reference ray changes parity. For this reason, NS lists the entry-exit (to and from the surface) intersection pairs to color the grid correctly. During this step, if one putative exit intersection is closer to its entry partner than 10^–7^ Å, NS currently discards it. In the new NS version, instead, we discard both the near entry-exit intersections, hence maintaining parity in the event that the parity was already present for that ray. We call the previous and current strategies “1-point skipping” and “2-point skipping” respectively. We found out that this new heuristic is more effective.

In these sporadic cases in which the in/out grid indicates that there is a sudden local change in its status along a cube edge and hence that there should be a vertex but there is none, a vertex should be created therein. In these cases, vertices and normals have to be approximated; specifically, a vertex is created in the middle of the pertinent cube edge during the MC stage. At the end of the MC stage and the triangulation, we reconstruct the normals of the previously dangling vertices by averaging over the normals of the neighboring triangles. Here, we do not employ a brute algorithm and fully sized intermediate buffers. In particular, we allocate a buffer to memorize neighboring triangle information solely for the added vertices and we flag these with a compressed buffer. Then, for each added vertex only we store the index of the neighboring triangles thanks to another partially full intermediate buffer. Last, we determine the normals of the added vertices by means of the neighboring triangles’ list and deallocate the two aforesaid intermediate buffers. In the results section we call this stage “normals’ approximation” (NA) stage.

## Results

Here we show how the new version of NS confirms its accuracy and allows us to handle higher grid resolutions and significantly larger molecular systems on commodity hardware. We applied minor code modifications to NS previous version 0.8 so as to extend its grid allocation limits and enable memory usage monitoring; this ensures a fair comparison between the software versions.

In a first set of experiments we checked that the newly developed code matches the accuracy of the previous version by considering over 1500 molecular structures including proteins, protein complexes and nucleic acids. Moreover, we checked that the new parallel build-up does not introduce approximations or artifacts. Additionally, we compared the NS’s performance with that given by the recent innovative machine learning based method Con2SES.^16^

In a second group of tests, we measured the enhanced capability of triangulating at very high resolutions. We show that rather large systems can be triangulated on a laptop equipped with 32 GB of RAM and an 8-core Intel i7. As some runs carried out by NS v0.8 at very high resolutions fail due to memory limits, we rerun experiments through the use of a CPU-only node equipped with two AMD EPYC 7713 2.0 GHz CPUs and 512 GB of RAM, which is part of the IIT Franklin HPC system. Next, we test the weak scaling capabilities for large atom counts via the H1N1 virus and check the strong scaling behavior. Last, we show the improved performance in pocket detections and the accuracy in supporting the solution of the Poisson–Boltzmann equation.

### Accuracy Validation

We took advantage of the data set of structures released with the Con2SES method^16^ to test the accuracy of the new NS version. It comprises 365 nucleic acids, 623 protein complexes and 574 proteins. We first built corresponding Amber topologies via tLeap in AmberTools and next exported to pqr files to get the radii. These were in turn converted to xyzr files, the input format used by NS (the new version can also read pqr files).

We checked the distribution of the signed percentage error of surface areas and volumes to validate the new NS version. Basically, the surface area distribution is a delta of Dirac centered at 0 (see Figure 5). We observed a considerably peaked Gaussian distribution centered at 0 in the case of the volumes. The discrepancies are hence unbiased and rather small, with the worst unsigned percentage given by a 3σ error of 0.3*%* for nucleic acids. This small, yet nonzero discrepancy, is likely due to the slightly more accurate way in which volumes are estimated with the NS v1.5. Indeed, NS v0.8 averages over three volume integrals (one per direction by considering rays passing on cubes edges), whereas NS v1.5 takes also advantage of grid rays (rays passing in the middle of grid cubes), thus averaging over four estimates.

**Figure 5:**
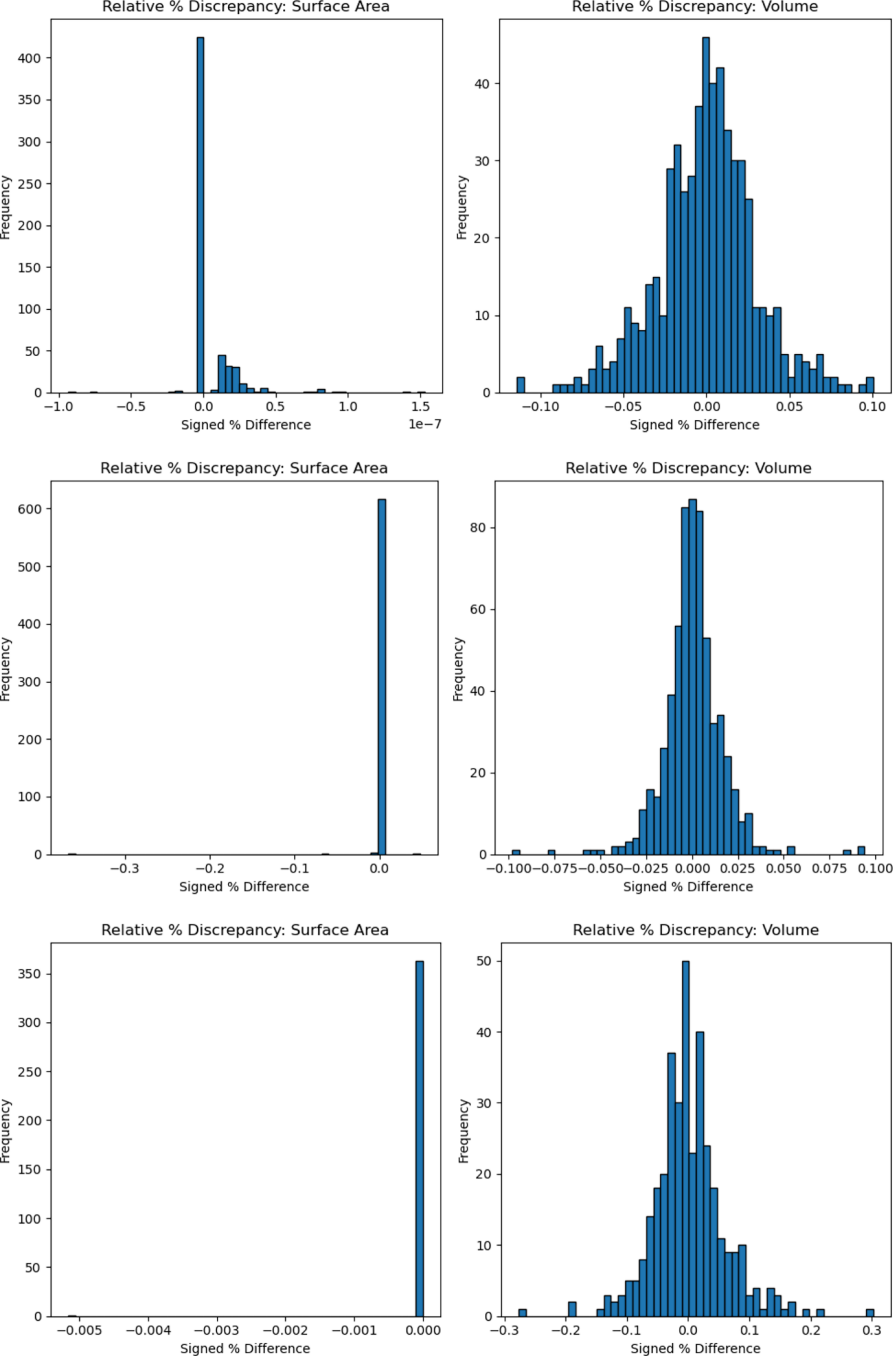
Signed percentage relative area and volume error differences between NS v0.8 and NS 1.5.

Last, we checked the reliability and accuracy of the parallel build-up algorithm. We empirically verified that for a probe radius ≤ 2 Å a halo layer thickness of 9 Å usually engenders correct results, as shown in the 21-system plot of Figure 6. By *correct* we mean that we obtained the same patch numbers and types, and reproduced the same six most significant digits of the area and volume estimations obtained with the single-thread runs. We found out that, for instance, a value of 8Å generates correct results, yet discrepancies may affect the volume and area results in the fifth significant digits. The H1N1 virus (see below text for more details) represents an exception: 5 most significant digits of the volume and area estimations are kept identical to the single thread and slab build-up run only if the halo layer thickness is 11 Å. This should not be surprising as geometry processing can be characterized by a considerable sensitivity since the order of operations suffer from roundoff errors and parallel build-up entails considering per-slab atomic systems which are completely different from the originally inputted atomic system (e.g., floating point additions are not associative and spatial subdivisions are spatially dependent intertwined problems). Our conservative parametrization relies hence on a default halo layer thickness of 12 Å (if needed the user can feed a different value from the configuration file) and allows to reproduce correct results even for large atomic systems such as the H1N1 virus and with the benefit of multithreading.

**Figure 6:**
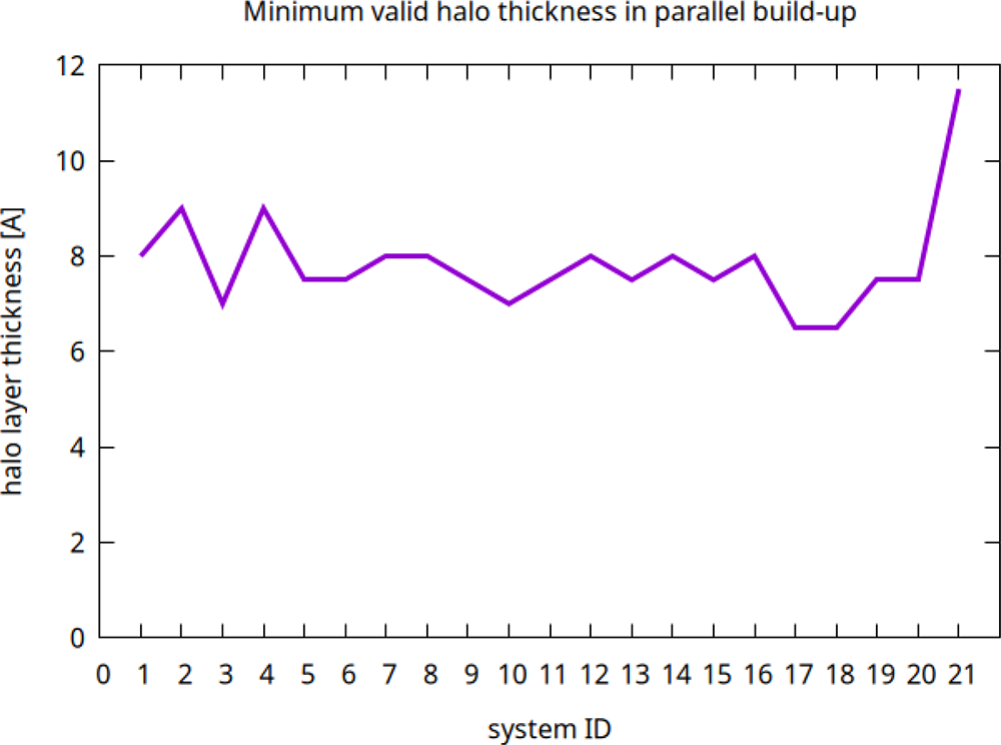
Minimum halo layer thickness guaranteeing wholly correct results for 21 molecular systems, including the ribosome, 1VSZ, and H1N1 (system IDs in the plots 1, 2, and 21, respectively). The results were obtained by choosing integer thickness values and halfway through values.

### Comparison with Con2SES-3D

Recently, methods have appeared which recast the problem of molecular surface computation to a machine learning one.^16,17^ They approximate the molecular surface via neural networks capable of estimating the scalar field implied in the molecular surface construction. Specifically, the recent Con2SES-3D^16^ was found to be particularly fast. This method is grid-aware and leverages GPU capabilities. Here, the results of the Con2SES-3D paper^16^ are compared with those given by the two versions of NS (see Table 1).

**Table 1:** Computing Time Comparison between Con2SES-3D and NS^a^

	nucleic acid	protein	protein complex
	avg. time (s)	avg. time (s)	avg. time (s)
Con2SES-3D	0.19 (0.13)	0.32 (0.18)	0.77 (0.87)
NS v. 0.8	0.18 (0.14)	0.29 (0.16)	1.23 (1.68)
NS v. 0.8*	0.10 (0.07)	0.16 (0.08)	0.71 (0.99)
NS v. 1.5	0.05 (0.03)	0.09 (0.05)	0.38 (0.49)
NS v. 1.5*	0.05 (0.03)	0.09 (0.05)	0.37 (0.48)

a We used the same datasets (columns) of Con2SES-3D as obtained from the corresponding GitHub project repository. Values are in seconds and represent average execution times, and standard deviations in parentheses. The results of Con2SES-3D are the best ones presented in^16^ where a NVIDIA GeForce RTX 4090 was used for the runs, the triangulation was not carried out, and the corresponding mesh was not saved. NS accomplished its full pipeline, including saving and smoothing the mesh. The results were obtained on an 8-core Intel i7 Ultra 258 V laptop CPU (no GPU). ”*” indicates running NanoShaper without a final memory clean up: a configuration keyword allows skipping it, which does not affect results and saves time in single-shot runs (useful only in API mode for repeated calculations).

We found out that NS v0.8 is slightly faster than Con2SES-3D when running on a recent Intel i7 258 V laptop processor when the final memory clean up is avoided (which does not affect the results and can be enabled by a keyword in the configuration file). We employed the data set available in the corresponding GitHub repository referenced by the Con2SES-3D manuscript. For this data set, the new NS version is between two and four times faster than Con2SES-3D. The effect of the presence of the cleanup on this NS version is not as impactful as for the version 0.8 since key memory data structures and buffers are more compactly stored and are deallocated drastically faster. We underline that the execution speeds are considerably higher despite the fact (a) that we do not employ a GPU, (b) that we triangulate, smooth and save the mesh, and (c) that we do not approximate the surface since all the computations are analytical so that the vertices are guaranteed to lie on the surface as per the SES technique.

### High-Resolution Triangulation

The first atomic system tested was the PDB entry 1VSZ [Fn fn1], the human adenovirus capsid (a 180 K atom system, after Hydrogen addition) the largest structure previously considered in ref 6, at grid resolutions *s* = 2, 4, 6, 8 Å^–1^. The second complex was a ribosomal unit comprising 377 K atoms, from the PDB entry 6R5Q, at grid resolutions *s* = 2, 4, 6 Å^–1^.

The performance results for 1VSZ are shown in Figure 7. Excessive memory requirement prevents NS version 0.8 from running at a scale greater than 6 Å^–1^ (N/A in the plot). At the scale equal to 2 Å^–1^(used commonly) the speed-up is about four and is consistently maintained at scale 4 Å^–1^. We run on the previously mentioned large RAM and core count computing node to fully assess the memory usage (Figure 8). We found that the NS version 0.8 is capable of handling the very high resolution of 8 Å^–1^ but the memory consumption is largely different. The new version is about 8 times less memory demanding in that it requires about 20 GB instead of about 160 GB. At the scale *s* = 2 Å^–1^ the memory required by version 1.5 is less than half of that demanded by the version 0.8.

**Figure 7:**
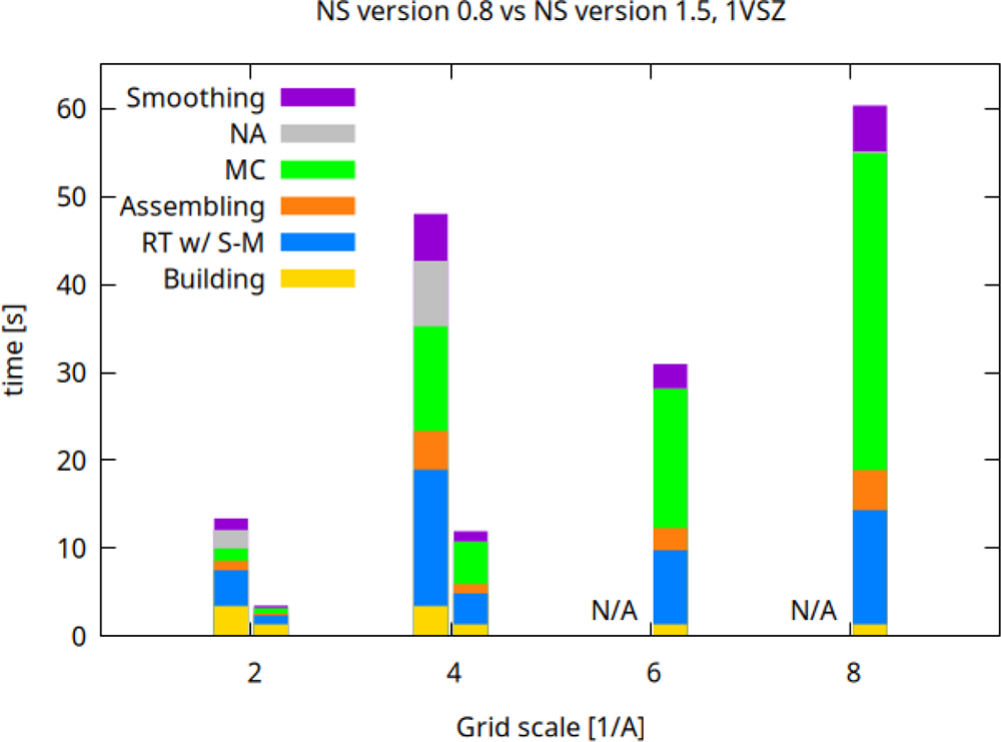
Comparison of the timings of the NS algorithmic steps of NS 0.8 and v 1.5 (left and right bars for each scale factor, respectively). The timings were obtained for the PDB entry 1VSZ (180 K atoms, after adding hydrogens) on a commodity laptop equipped with 32 GB of RAM and an Intel i7 with 8 cores and 16 threads. At scales larger than *s* = 4 Å^–1^, only version 1.5 is able to run due to the excessive memory requirements of version 0.8.

**Figure 8:**
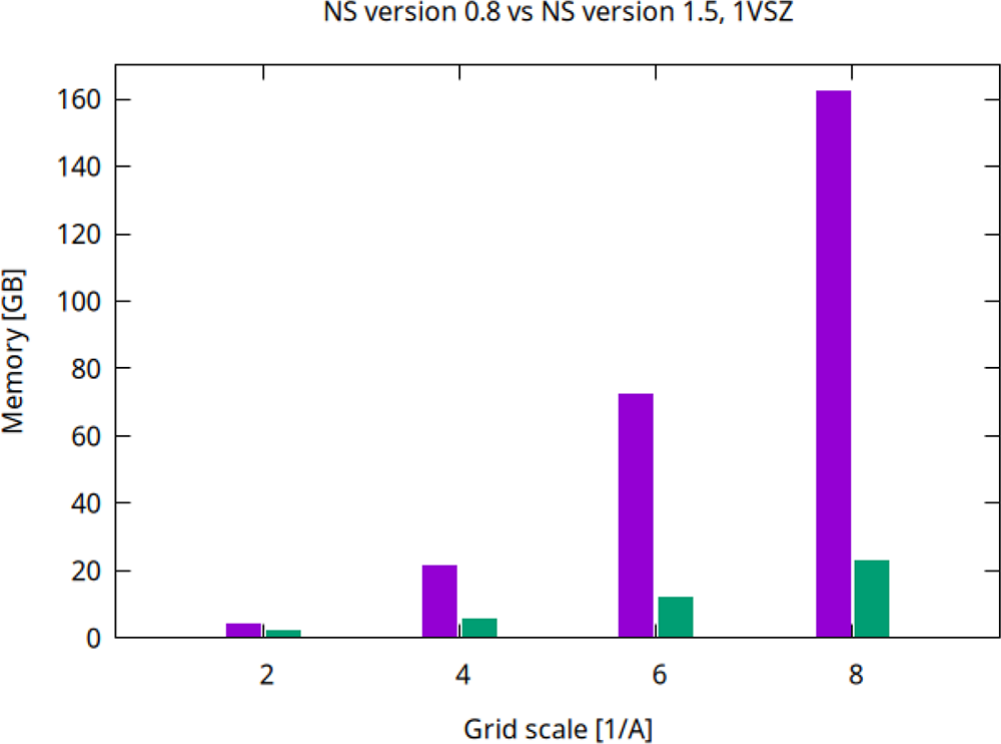
Peak memory of 1VSZ (180 K atoms, after adding hydrogens) as a function of *s* for version 0.8 (violet column for each scale factor) and version 1.5 (green column for each scale factor) on a 512 GB RAM node.

The second atomic system tested was a ribosome of 377 K atoms for which analogous results were obtained. At the familiar scale *s* = 2 Å^–1^, version 1.5 is 3.67 times faster (Figure 9). At the maximal scale *s* = 6 Å^–1^, a reduction in the memory usage of about 3 is achieved (see Figure 10).

**Figure 9:**
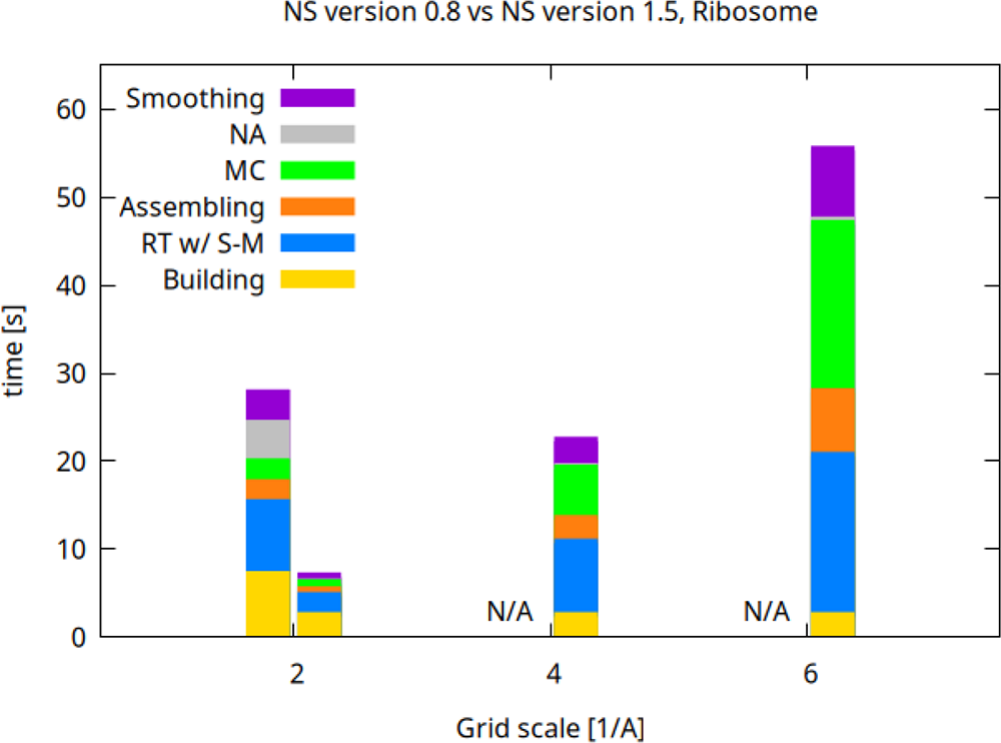
Comparison of the timings of the NS algorithmic steps of version 0.8 and version 1.5 (left and right bars for each scale factor, respectively). The timings were obtained for the Ribosome system (377 K atoms) on a commodity laptop equipped with 32 GB of RAM and an Intel i7 with 8 cores and 16 threads. Only version 1.5 is able to run at scales higher than *s* = 2 Å^–1^ because of memory restraints.

**Figure 10:**
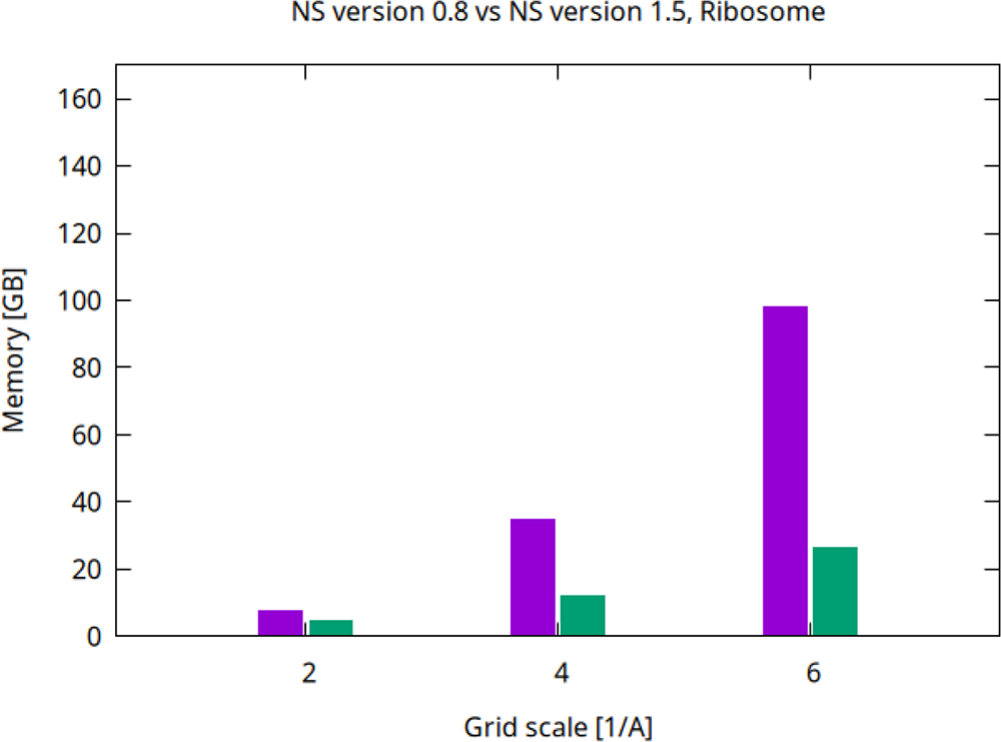
Peak memory for the ribosome (377 K atoms) as a function of *s* for version 0.8 and version 1.5 (violet and green bars, respectively) on a 512 GB RAM node.

Considering the processing stages separately, we found out that the build-up phase is about 2.6× faster for both the analyzed systems while the RT step delivers a ≈4× boost. The analytical ray-torus intersection routine contributes substantially to this speed increase. The runtime allocation and updating of the sparse, two-level status map, S-M, which is a grid storing the cavity and in/out information, takes around 35*%* of the RT time. Noteworthy, despite the more complicated data allocation and updating of the sparse and compressed layouts with respect to those of uniform grids, the RT and MC performances are not impacted significantly. The per-ray intersection sorting stage only takes about 0.36*%* of the total computing time. The assembling of the cross-thread intersection data is much faster when the new algorithm is employed; in particular, when *s* = 2 Å^–1^, it is 3.53 and 4.42 times faster than that of NS v0.8 for the ribosome and 1VSZ, respectively. The MC stage is now much more cache oblivious, and when *s* = 2 Å^–1^ it is also 2.80 and 2.37 times faster for the ribosome and 1VSZ, respectively. The NA stage devoted to estimating the normals of approximated vertices now takes an insignificant computing time; it also demands an insignificant memory since the substantial data allocation is only concerning the approximated vertices, which are often absent or insignificant in number. In particular, the new scheme takes an insignificant time relative to the previous algorithm, even when approximated vertices are added. In the case of the ribosome at *s* = 2 Å^–1^, the memory consumption of the assembling stage plus the MC and the NA steps is about 2 GB with NS v0.8 and about 0.8 GB with NS v1.5. The new smoothing algorithm is nearly five times faster than that of the NS v0.8. Taken together, these results show that it is possible to triangulate complexes with a fairly large number of atoms and the standard scale of *s* = 2 Å^–1^ by leveraging a modern, well-equipped laptop (32 GB of RAM, 8 cores).

### Triangulating Very Large Complexes

Here we present the capability of the new NS release in terms of weak scaling by checking time and memory needs as a function of the number of atoms. To this aim, we employed the swine flu virus H1N1, which comprises 6439 K atoms (we neglected the ≈8000 K Hydrogen atoms). We considered progressively larger subsets of this system to benchmark the scaling behavior of the new software in the various computational steps (see Figure 11) and compared it with that given by NS v0.8. We computed the barycenter and progressively expanded around it in four steps so as to extract the aforesaid atomic subsets and thus have 2310, 4332, 5475 and 6439 K atoms, respectively. The results were obtained using a ray grid resolution parameter *s* = 2 Å^–1^ and the previously mentioned large RAM node. The results show that the overall running time and each single phase scales linearly with the number of atoms as for NS v0.8 but with a significantly improved scaling constant. The computational boost is 12.5× in the case of the untrimmed atomic system. Furthermore, we assessed the scaling of the memory requirement of NS as a function of the number of atoms (see Figure 12). The results confirm that the memory consumption scales proportionally to the number of atoms, but is characterized by significant reductions when the new NS version is utilized. When taking into account the full atomic system, the memory is reduced about three times.

**Figure 11:**
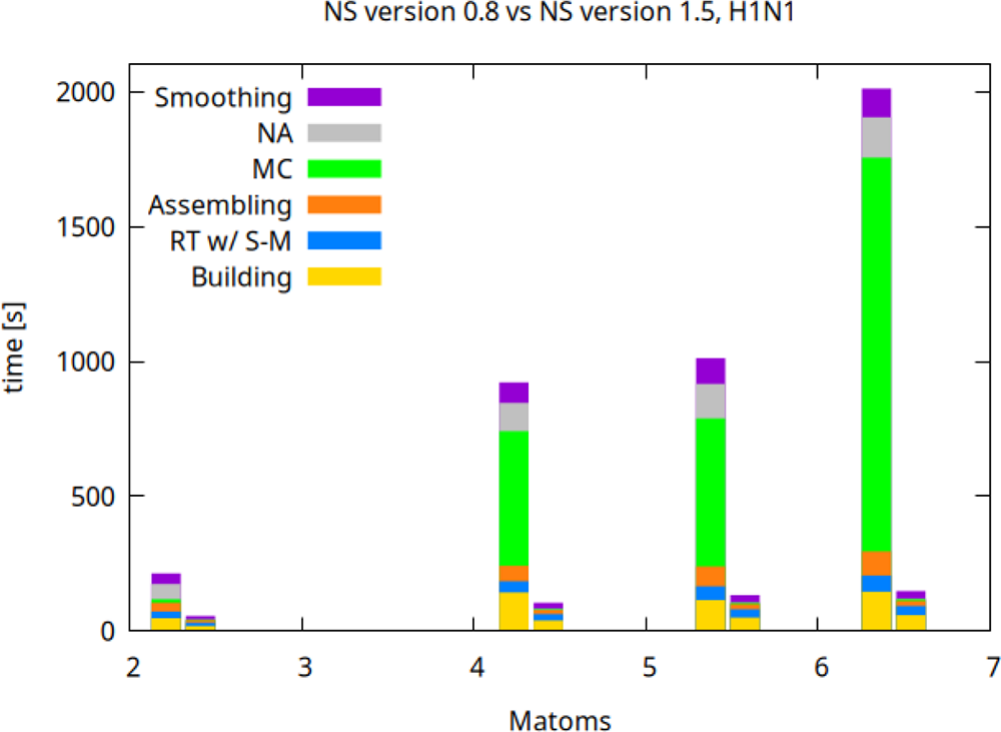
Scaling of the computing time of the specific NS computational steps as a function of the number of millions atoms (Matoms) of the H1N1 system for version 0.8 vs 1.5 (left and right bars for each tested subset of atoms). The algorithmic steps are SES building phase, ray tracing (RT), assembling of intersections and normals’ information from the data private to the threads employed during the RT, Marching Cubes (MC), and normals’ approximation stage (NA).

**Figure 12:**
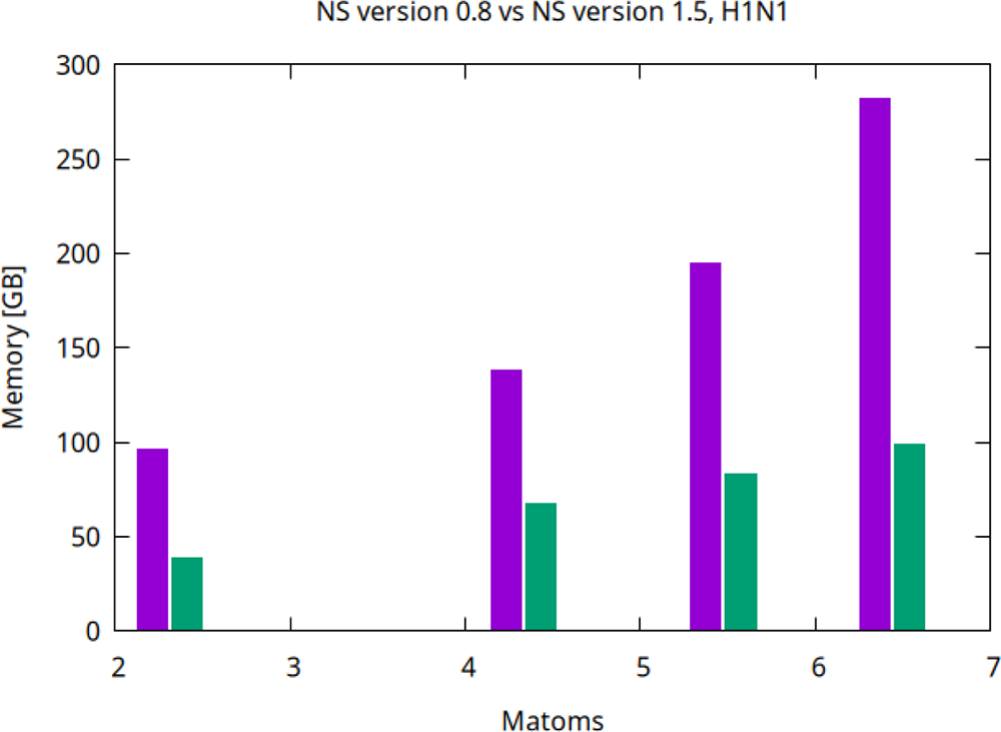
Peak memory as a function of the number of atoms within increasingly larger subsets of the H1N1 complex for NS version 0.8 (violet bar) and version 1.5 (green bar).

In detail, for this system, the build-up speed-up is analogous to that featured by the previous tests (about 3×). The impact of the analytical ray-torus intersection routine is still significant: for the full virus the RT of the new software is around 30*%* slower if the iterative intersection algorithm is utilized in place of the analytical routine. During RT, the runtime S-M allocations and updates take around 40*%* of the RT phase; this overhead is unavoidable and in fact a 30*%* overhead was noted when updating the uniform grid of smaller systems. In the case of the untrimmed virus, the assembling stage, the MC, the NA step and the smoothing one were sped up by 4.73×, 235×, 179×, and 3.61×, respectively. Last, we mention that we were able to triangulate up to 1.3 milion atoms of this system on the previously employed 32 GB RAM, 8 cores laptop.

### Multithreading Scalability

The multithreading scalability was assessed by runs executed using the ribosome and a 7817-atoms system on a laptop. Figure 13 shows the overall multithreading performance scalability whereas the SI shows the timing behavior of the specific multithreaded steps, e.g., RT and MC. The overall strong scaling is significantly improved since more steps are now parallel. The plot shows that there is both a considerable boost even for the single-thread case and the overall scaling is also improved when the system size versus thread count is not too unfavorable. Note that the build-up phase, despite being parallel, is not particularly cache friendly (because the threads deal with completely disjointed atomic and patch sets) and hence not very multithreading friendly, and that the smoothing stage, albeit considerably boosted in the new version, is not parallel. This limits the overall scalability, which instead is very effective in the cases of the RT and of the MC phases (see SI).

**Figure 13:**
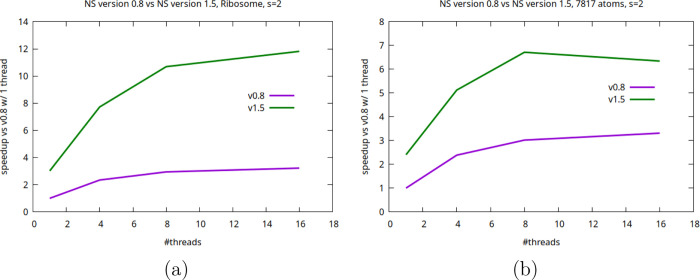
Multithreading scalability of NS version 0.8 and version 1.5 (violet and green lines, respectively) for (a) ribosome (377 K atoms) and (b) 7817-atoms system at *s* = 2 as a function of number of threads on a commodity laptop equipped with 32 GB of RAM and an Intel i7 with 8 cores and 16 threads. The plot reports speed-up factors relative to the single-thread runs of the NS version 0.8. When using version 1.5 with 8 and 16 threads to analyze the smaller system the number of slabs and threads to carry out the build-up stage was 6 due to system size and halo layer restrictions.

### Pocket Detection

The determination of the pockets is a very demanding task because it entails not only detecting the pockets but also triangulating each one.^6^ The new NS version maintains the current pocket detection technique, yet inherits the previous accelerated steps. During pocket detection the S-M is continuously read and written, hence its bilevel form is not particularly efficient. For this reason a plain 3D S-M grid is advisible for this task and it is set as default. When a uniform S-M is employed NS v1.5 is 47% and 40% faster than v0.8 when determining the pockets of the ribosome and of 1VSZ, respectively.

### Poisson–Boltzmann Equation Solution

NS is also employed as a library within the Poisson–Boltzmann solver NextGenPB^18^ to compute the molecular surface for the evaluation of the electrostatic properties of macromolecules in solvent. In this solver, NS provides both the intersection points and their normal vectors to the molecular surface. NextGenPB uses this information to distinguish between the interior and exterior regions of a molecule. Moreover, such an analytical information has been employed to improve the accuracy of the computed electrostatic potential and energy. For this reason, we compared the polarization energy results obtained by NextGenPB when coupled with NS v0.8 and with v1.5, using the Con2SES data set. In agreement with the results reported for the total molecular surface area – computed using the same intersections and rays employed by NextGenPB—the results obtained with the two software versions are virtually identical. In fact, the maximum absolute error is 0.023 *k*_B_ *T*, and the maximum relative error is 0.0006%. All NextGenPB calculations were performed using a grid spacing of 0.5 Å, with a 95% per-fill ratio, and a derefinement method applied up to 20% per-fill to impose homogeneous Dirichlet boundary conditions.

## Conclusions

This manuscript presents a significantly faster and memory-parsimonious evolution of NS. We parallelized many parts of the code, made cache friendly several routines, employed very lightweight data structures, added a faster and more accurate analytical ray-torus intersection routine and chiefly redesigned the ray tracing phase via a patch-based approach. All these modifications allowed us to deal with hundreds of thousands of atoms on a commodity laptop and to triangulate the surface of an entire virus featuring 6.5 million atoms on a cluster node while only utilizing 100 GB of RAM.

For future development purposes, we envision an MPI version. All the main steps of the SES computation in NS are trivially parallel so we expect to naturally embody them into an MPI implementation with modest interprocess communications. The MPI implementation would target not only increased performance but mainly the capability of distributing the memory load across nodes, thereby allowing to triangulate huge atomic systems.

## Supplementary Material



## Data Availability

NanoShaper is available at https://gitlab.iit.it/SDecherchi/nanoshaper/. We provide a python script to support the compilation (setup.py), a Docker recipe and a user guide.

## Footnotes

1 At present, the 1VSZ structure has been replaced in the PDB repository by the 4CWU one.

## Notes

Usage of NanoShaper; patch-based ray tracing; quartic polynomial root finding; cubic equation solver; bilevel grid cells allocation and update; solvent-excluded surface intersections accuracy; and multithreading scalability (PDF)
