# Supplementary material for: Patch-Based Ray Tracing in NanoShaper Boosts Molecular Surface Computation
Source: J Chem Inf Model. 2025 Dec 23;66(1):461–71. doi: 10.1021/acs.jcim.5c02287 (PMC12801300; doi:10.1021/acs.jcim.5c02287)
Supplement: Supplementary file 1 [file ci5c02287_si_001.pdf]

Supplementary Information: Patch-Based Ray Tracing in NanoShaper Boosts Molecular Surface Computation

Marco Domenico Mazzeo,[†] Vincenzo Di Florio,[†,‡] Walter Rocchia,[*,†] and Sergio Decherchi[*,¶]

†*Concept Lab, Fondazione Istituto Italiano di Tecnologia, via Morego 30, 16163 Genoa, Italy*

‡*MOX Laboratory, Department of Mathematics, Politecnico di Milano, Piazza Leonardo Da Vinci, 32, Milano, 20133, Italy*

¶*Data Science and Computation Facility, Fondazione Istituto Italiano di Tecnologia, via Morego 30, 16163 Genoa, Italy*

E-mail: walter.rocchia@iit.it; sergio.decherchi@iit.it

Usage of NanoShaper

NS computes the triangulation of the surface of a molecular system and estimates its volume and area. Additionally find pockets/cavities and triangulate them. It is also possible to interface it via its new API to a Poisson-Boltzmann solver, for example to NextGenPB (see main text). Here we provide some further technical details also available in the user guide in the git repository.

NS can be configured and compiled with the repository script *setup.py* in Linux or Unix (and hence in Mac), or can be compiled and installed manually. It can be compiled as a

standalone executable or a library (.so file, API).

Running NS entails launching its executable after compilation of its configuration file. The configuration file specifies all settings including the atomic system file which is in xyzr format (coordinates plus radius). The current version also supports loading directly PQR files directly. The triangulation can be saved in different formats, including the MSMS one; by default the .off format (or .off plus normals) is used.

Details on patch-based ray tracing

Here we explain the data structures utilised to handle the patch-based algorithm. All the ray-patch intersections are computed and stored in a buffer of vectors, called **patch_intersec**[ ][ ], where a patch index involves the left dimension and an intersection is indexed by the right dimension. Another buffer of vectors, **patch_pixel**[ ], stores the pixel identifier for each intersection. After filling it during the RT, we use **patch_intersec**[ ][ ], and **patch_pixel**[ ][ ] to build a two-dimensional screen-wise buffer, named **screen_intersec**[ ][ ], which stores for each pixel all the intersections of the patches behind that pixel. This is carried out by simply looping through the patches, accessing the data stored in **patch_intersec**[ ][ ] and copying them in **screen_intersec**[ ][ ]. **screen_intersec**[ ][ ] is needed because all the per-pixel computations have to be performed during the fifth step of the patch based method mentioned in the main text.

Quartic polynomial root finding

Here, a simplified pseudo-code for the solution of a quartic equation required by the torus-ray intersection is provided (variable initialization is omitted). We assume a monic polynomial wherein the leading coefficient $a = 1$:

Algorithm: Compute the real solutions of a quartic equation

Input: Coefficients b, c, d, e

Output: Real roots stored in roots[4] and the number of real solutions num_sol

- Compute intermediate values:
  - $b_2 = 0.5b$
  - $b_4 = 0.25b$
  - $p = c - 1.5(b_2 \cdot b_2)$
  - $q = d + b_2(b_2 \cdot b_2 - c)$
  - $r = e + b_4(b_4(c - 3b_4 \cdot b_4) - d)$
  - $f = p \cdot p - 4r$
  - $\delta = 4f(4r(p \cdot p) - p * (q \cdot q) - 16. * (r \cdot r)) + (q \cdot q) * (128p \cdot r - 27(q \cdot q)$
- **If** $e \neq 0$ **and** ($|p|$ **or** $|q|$ **or** $|r|$ **or** $|\delta|$ is greater than 3000), change variable:
  - $d_{su2e} = 0.5\frac{d}{e}$
  - Update coefficients:
    * $p = -1.5d_{su2e}^2 + \frac{c}{e}$
    * $q = d_{su2e}(d_{su2e}^2 - \frac{c}{e}) + \frac{b}{e}$
    * $r = d_{su2e}\left(-\frac{3}{16}d_{su2e}^3 + 0.25d_{su2e}\frac{c}{e} - 0.5\frac{b}{e}\right) + \frac{1}{e}$
  - Recompute f and δ
- Determine the number of real solutions:
  - **If** $\delta < 0$ **or** ($\delta = 0$ **and** $p \geq 0$ **or** $f < 0$), **then set** $\text{num\_sol} = 2$
  - **Else if** ($p < 0$ **and** $f > 0$) **or** ($\delta = 0, p = 0, r = 0$) **then set** $\text{num\_sol} = 4$
  - **Else set** $num\_sol = 0$ and **return**
- Compute m as the real root of the cubic equation:
  - $m = \text{realCubicSol}(p, 0.25p^2 - r, -0.125q^2)$

- **If** $m \leq 0$, **then set** $num\_sol = 0$ and **return**

- Compute solutions:
  - Compute intermediate values:
    - **If** $m < 10^{-14}$
      - $p_1 = 2p$
      - $p_2 = q \cdot q/f$
      - $p_3 = 2\sqrt{f}$
      - $sol\_delta\_max = p3 - p_1 - p_2$
      - $coeff = 2|q|/f$
    - **Else**
      - $p_1 = -2(p + m)$
      - $p_2 = \sqrt{2q \cdot q/m}$
      - $sol\_delta\_max = p_1 + p_2$
      - $coeff = \sqrt{2m}$
  - Compute first two roots:
    - $roots[0] = 0.5((q \geq 0\ ?\ -coeff : coeff) - \sqrt{sol\_delta\_max})$
    - $roots[1] = 0.5((q \geq 0\ ?\ -coeff : coeff) + \sqrt{sol\_delta\_max})$
  - **If** $num\_sol = 4$:
    - **If** $m < 10^{-14}$ **Then** $sol\_delta\_min = -p_1 - p_2 - p3$
    - **Else** $sol\_delta\_min = p_1 - p_2$
    - $roots[2] = 0.5((q \geq 0\ ?\ coeff : -coeff) - \sqrt{sol\_delta\_min})$
    - $roots[3] = 0.5((q \geq 0\ ?\ coeff : -coeff) + \sqrt{sol\_delta\_min})$

- Adjust solutions based on transformation:
  - **If** variable change occurred **then set** $roots[i] = \frac{1}{roots[i] - 0.25d/e}\ \forall i$

- **Else set** $roots[i] = roots[i] - 0.25b \ \forall i$

- **Output:** Roots of the quartic equation $roots[i] \ \forall i$

Cubic equation solver

Here, we provide a simplified pseudo-code of our cubic equation solver implemented in the function realCubicSol(b, c, d) wherein b, c, d are the coefficient of the polynomial and $cbrt()$ is the C function which returns the cube root of a number.

Algorithm: Compute the largest real root of a cubic polynomial

Input: Coefficients b, c, d

- $p = -(1/3)b \cdot b + c$
- $q = (2/27)b \cdot b \cdot b - (1/3)b \cdot c + d$
- $D3p = (1/27)p \cdot p \cdot p$
- $D3 = D3p + 0.25q \cdot q$
- **If** $|c| < b^2 \cdot 10^{-6}$ **and** $|d| < |b| \cdot 10^{-6}$:
  - **Return** $c/b - b - d/b^2$
- **Else if** $|d/c| < 4 \cdot 10^{-4}$:
  - $r = d/c$
  - **If** $D_3 \leq 0$ **then return** $0.5(r - b + \sqrt{(b-r)(b-r) - 4(c - r * (b-r))})$
  - **Else return** $-r \cdot r(b-r)/(3r \cdot r - 2br + c) - r$
- **Else**:
  - **If** $D_3 \geq 0$:

- $radD3 = \sqrt{D3}$
- $A = cbrt(radD3 - 0.5q)$
- $psu3A = cbrt(radD3 + 0.5q)$
- **Return** $A - psu3A - (1/3) * b$

– **Else**:

- $Aarg = \arccos(-0.5q/\sqrt{-D3p})$
- **Return** $2\sqrt{-(1/3) * p}\ \cos((1/3) * Aarg) - (1/3)b$

Bilevel grid cells allocation and update

Algorithm: Update the bilevel grid $bg[\ ][\ ]$

Input: Input value val, coordinates of the voxel (i, j, k), number of voxels (N_x, N_y, N_z); long integers are employed where appropriate.

Data updating: write a value in the 4^3-voxel mini-grid (to be allocated, if needed) of the spanned coarse cell

- **If** value to write is equal to the background value, **Then** skip the rest
- Compute the coarse cell coordinates via shifting: $i^{coarse} = i >> 2$; $j^{coarse} = j >> 2$; $k^{coarse} = k >> 2$
- Computation of coarse grid size: $N_x^{coarse} = N_x >> 2$; $N_y^{coarse} = N_y >> 2$
- Make sure the coarse grid is large enough if sides are not multiple of 4:
- **If** $(N_x^{coarse} << 2) < N_x$, **Then** $++N_x^{coarse}$
- **If** $(N_y^{coarse} << 2) < N_y$, **Then** $++N_y^{coarse}$
- $ID^{coarse} = (k^{coarse} N_y^{coarse} + j^{coarse}) N_x^{coarse} + i^{coarse}$

- If the mini-grid is not allocated, **If** $bg[ID^{coarse}] == NULL$, **Then** allocate $bg[ID^{coarse}][*]$ and set all its elements equal to the background value
- Compute coordinates and identifier of the voxel within the current mini-grid via fast operators
  - $i^{fine} = i - (i^{coarse} << 2)$
  - $j^{fine} = j - (j^{coarse} << 2)$
  - $k^{fine} = k - (k^{coarse} << 2)$
- $ID^{fine} = (k^{fine} << 4) \mid (j^{fine} << 2) \mid i^{fine}$
- Write the input value in the two-level grid: $bg[ID^{coarse}][ID^{fine}] = val$

Solvent Excluded Surface intersections accuracy

This section presents the intersections accuracy results given by the iterative intersection algorithm (IIA) implemented in the NS version 0.8 and the new analytical intersection algorithm (AIA, implemented in NS version 1.5). Also we evaluate the approaches we employed to manage limit cases, dubbed "1-point skipping" (1PS) and "2-point skipping" (2PS) discussed in the main text. In Tables 3, 2 and 1 the accuracy increase given by the analytical torus-intersection algorithm over the iterative method of the previous NS version is notable when analyzing 1VSZ since the maximal number of approximated rays remains one while increasing the grid resolution parameter s. The combination of this algorithm with the 2-point skipping approach further decreases the number of approximations to carry out; indeed, the number of approximated rays due to wrong parity check are zero in all the cases, and the number of approximated intersections (to tackle inconsistent grid information) in the case of the ribosome is around one third with respect to the other algorithmic combinations. This result is confirmed for the virus case.

Table 1: Number of approximated rays and intersections (in parentheses) when using the NS version 0.8, implementing the iterative ray-torus intersection algorithm (IIA) and 1-point skipping (1PS), and the version 1.5 with IIA and 1PS, or analytical ray-torus intersection algorithm AIA and 1PS, or the AIA and 2PS in the case of 1VSZ (180K atoms) as a function of the grid resolution s.

s	NS v. 0.8	NS v. 1.5 w/ IIA & 1PS	*v*. 1.5 w/ AIA & 1PS	*v*. 1.5 w/ AIA & 2PS
2	1(6)	1(6)	0(0)	0(0)
4	1(21)	1(12)	1(32)	0(12)
6	N/A	3(67)	0(0)	0(0)
8	N/A	5(157)	1(8)	0(0)

Table 2: Number of approximated rays and intersections (in parentheses) when using the NS version 0.8, implementing the IIA and 1PS, and the version 1.5 with IIA and 1PS, or AIA and 1PS, or AIA and 2PS in the case of the ribosome (377K atoms) as a function of the grid resolutions$s$.

s	NS v. 0.8	NS v. 1.5 w/ IIA & 1PS	v. 1.5 w/ AIA & 1PS	v. 1.5 w/ AIA & 2PS
2	24(1021)	24(1374)	24(1374)	0(0)
4	N/A	113(6763)	111(6653)	0(0)
6	N/A	238(14486)	236(14416)	0(0)

Table 3: Number of approximated rays and intersections (in parentheses) when using the NS version 0.8, implementing the IIA and 1PS, and the version 1.5 with AIA and 2PS as a function of the number of atoms through increasingly larger subsets of the H1N1 complex.

Matoms	NS v. 0.8	NS v. 1.5 w/ AIA & 2PS
0.663	2(52)	0(0)
2.31	270(19674)	0(5355)
4.33	1176(70446)	0(19986)
5.38	589(35803)	0(3800)
6.34	438(20213)	0(10521)

Multithreading scalability

The multithreading behaviour of the various computational stages was assessed by runs executed using the ribosome and a 7817-atom system on a i7 laptop. Fig. 1 shows thread-wise timing behaviour of the specific NS stages, *e.g.*, RT and MC.

Results show that NS heavily benefits of the parallel build-up only for rather big systems (like the Ribosome). Instead the other stages, particularly marching cubes and ray tracing, benefit of parallelization even for relatively small systems.

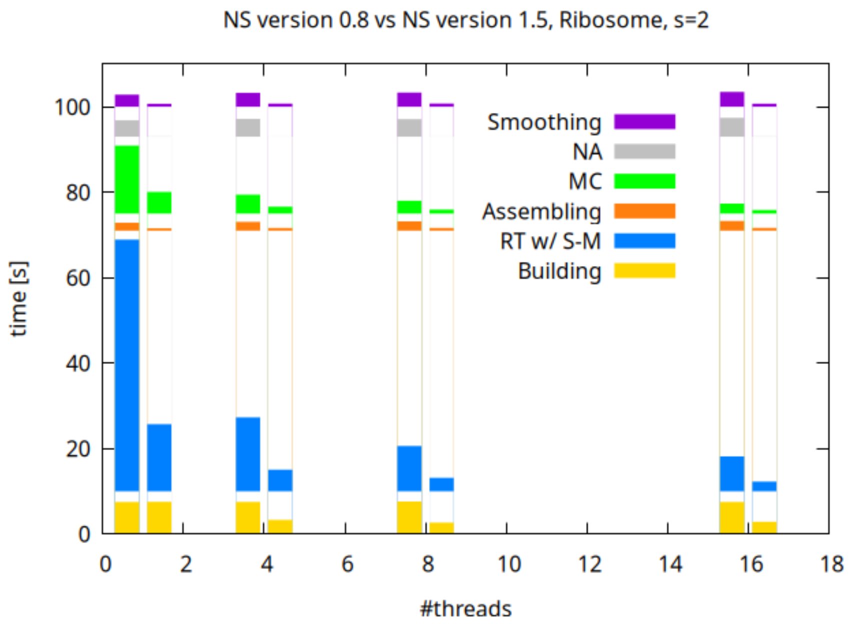

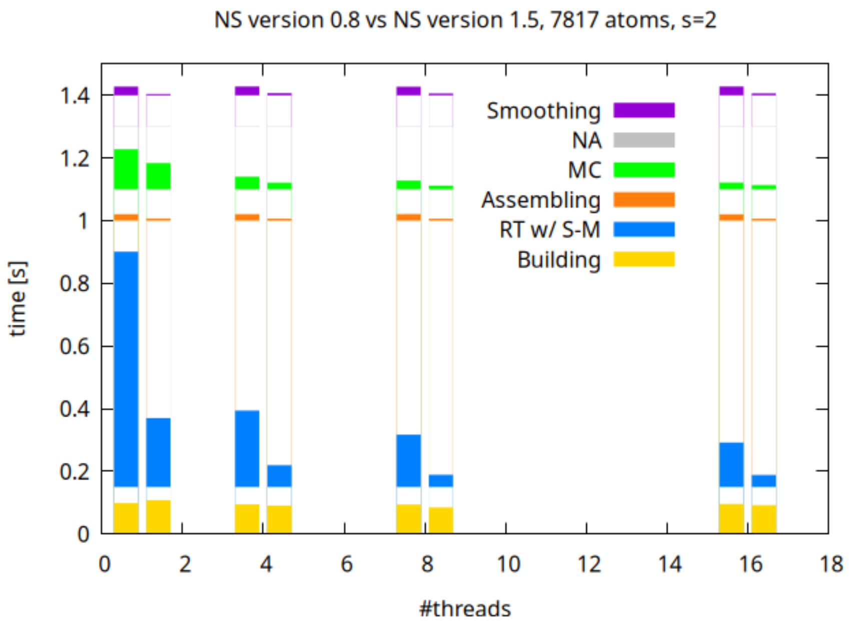

(a)

(b)

Figure 1: Multithreading behaviour of the NS algorithmic steps (build-up, RT, assembling, MC, normal approximations and smoothing) of NS version 0.8 and version 1.5 for (a) the ribosome (377K atoms) and (b) a 7817-atom system at $s = 2$ as a function of number of threads on a commodity laptop with 32 GB of RAM and an Intel i7 with 8 cores and 16 threads. When using version 1.5 with 8 and 16 threads to analyse the smaller system the number of slabs and threads to carry out the build-up stage was automatically set 6 due to system size and halo layer restrictions.
